# Genome-Wide Association Study for Grain Micronutrient Concentrations in Wheat Advanced Lines Derived From Wild Emmer

**DOI:** 10.3389/fpls.2021.651283

**Published:** 2021-05-14

**Authors:** Jia Liu, Lin Huang, Tingxuan Li, Yaxi Liu, Zehong Yan, Guan Tang, Youliang Zheng, Dengcai Liu, Bihua Wu

**Affiliations:** ^1^State Key Laboratory of Crop Gene Exploration and Utilization in Southwest China, Sichuan Agricultural University, Wenjiang, China; ^2^Triticeae Research Institute, Sichuan Agricultural University, Wenjiang, China; ^3^College of Resources, Sichuan Agricultural University, Wenjiang, China; ^4^Key Laboratory of Crop Genetic Resources and Improvement, Ministry of Education, Sichuan Agricultural University, Ya'an, China

**Keywords:** wild emmer, common wheat, grain micronutrient concentrations, wide hybridization, GWAS, biofortification

## Abstract

Wheat is one of the important staple crops as the resources of both food and micronutrient for most people of the world. However, the levels of micronutrients (especially Fe and Zn) in common wheat are inherently low. Biofortification is an effective way to increase the micronutrient concentration of wheat. Wild emmer wheat (*Triticum turgidum* ssp. *dicoccoides*, AABB, 2n = 4x = 28) is an important germplasm resource for wheat micronutrients improvement. In the present study, a genome-wide association study (GWAS) was performed to characterize grain iron, zinc, and manganese concentration (GFeC, GZnC, and GMnC) in 161 advanced lines derived from wild emmer. Using both the general linear model and mixed linear model, we identified 14 high-confidence significant marker-trait associations (MTAs) that were associated with GFeC, GZnC, and GMnC of which nine MTAs were novel. Six MTAs distributed on chromosomes 3B, 4A, 4B, 5A, and 7B were significantly associated with GFeC. Three MTAs on 1A and 2A were significantly associated with GZnC and five MTAs on 1B were significantly associated with GMnC. These MTAs show no negative effects on thousand kernel weight (TKW), implying the potential value for simultaneous improvement of micronutrient concentrations and TKW in breeding. Meanwhile, the GFeC, GZnC and GMnC are positively correlated, suggesting that these traits could be simultaneously improved. Genotypes containing high-confidence MTAs and 61 top genotypes with a higher concentration of grain micronutrients were recommended for wheat biofortification breeding. A total of 38 candidate genes related to micronutrient concentrations were identified. These candidates can be classified into four main groups: enzymes, transporter proteins, MYB transcription factor, and plant defense responses proteins. The MTAs and associated candidate genes provide essential information for wheat biofortification breeding through marker-assisted selection (MAS).

## Introduction

Mineral elements are essential micronutrients for animals and human beings. It is estimated that approximately half of the world's population are at risk of micronutrient deficiency (Gregory et al., 2017; Tabbita et al., 2017), resulting in serious health problems (Cakmak and Kutman, 2018). For example, the deficiency in iron (Fe) and zinc (Zn) may cause overall poor health, anemia, increase morbidity and mortality rates, and low worker productivity (Peleg et al., 2008; Gibson, 2012; Borrill et al., 2014), whereas the manganese (Mn) shortfall may affect reproductive ability, mental development, and bone development (Li and Tan, 2012). In addition, the previous study showed that crops grown in high CO_2_ environments not only have low nutritional value, but also lack Fe, Zn, and other important micronutrients (Myers et al., 2014). Developing micronutrient-enriched agricultural crops (biofortification), agronomically and/or genetically, is considered the most cost-effective and sustainable approach to alleviate malnutrition and related health problems (Peleg et al., 2008; Bouis and Saltzman, 2017).

Wheat (*Triticum aestivum*, 2n = 6x = 42, AABBDD) is a major staple food crop worldwide. Therefore, the composition and nutritional quality of wheat grains have a significant impact on human health and well-being. Modern wheat cultivars are, however, inherently low in micronutrients and show a narrow genetic variation for micronutrients to be exploited in breeding programs (Velu et al., 2017). One effective strategy to improve the amount of mineral elements in wheat grains is to exploit the “left behind” genetic variation in wild relatives for grain micronutrients (Peleg et al., 2008, 2009).

Wild emmer wheat (*Triticum turgidum* ssp. *dicoccoides*, 2n = 4x = AABB) is the tetraploid progenitor of cultivated wheat, harboring a rich allelic repertoire for improvement of various economically important traits in wheat (Gomez-Becerra et al., 2010), including micronutrient concentrations (Yan et al., 2018). Substantially high concentrations of up to 190 mg kg^−1^ for Zn and 109 mg kg^−1^ for Fe in grains of wild emmer have been reported (Cakmak et al., 2004, 2010).

The accumulation of minerals in seeds depends on a plethora of processes that are controlled by several genes (Peleg et al., 2009). Till now, dissection of the genetic basis of grain micronutrients content in wild emmer wheat was performed using tetraploid wheat recombinant inbred lines (RILs), derived from a cross between durum wheat (*T. turgidum* ssp. *durum*, cv. Langdon) and wild emmer wheat (accession G18-16) (Peleg et al., 2009; Fatiukha et al., 2020). The introgression and identification of wild emmer quantitative trait locus (QTL) linked to micronutrient accumulation in a hexaploid wheat background have been less reported. The wild emmer gene *Gpc-B1* that belongs to the NAC-domain transcription factor affects grain protein content (GPC), grain Zn, Fe, and Mn concentrations (GZnC, GFeC, and GMnC) in wheat (Uauy et al., 2006; Distelfeld et al., 2007). However, this gene was associated with reductions in grain weight and yield in some environments and wheat cultivars (Uauy et al., 2006; Brevis and Dubcovsky, 2010; Tabbita et al., 2013). Thus, the exploration of genomic regions associated with high GZnC, GFeC, and GMnC that have less negative effect on grain yield is desirable.

Genome-wide association analysis (GWAS) has been widely used for deciphering the genetic basis of multiple traits in crops (Su et al., 2016; Wang et al., 2016; Ates et al., 2018; Liu et al., 2019). It was available to study QTLs related to agronomically important traits in large sets of germplasm resources such as landraces (Liu et al., 2017), elite cultivars (Sukumaran et al., 2015), and advanced breeding lines (Wang et al., 2017) as well as backbone parents and their derived lines (Yu and Tian, 2012; Yu et al., 2014; Xiao et al., 2016; Liu et al., 2019). In wheat, GWAS has been extensively applied to reveal genomic regions controlling traits such as GPC (Liu et al., 2019), grain ionome (Fatiukha et al., 2020), and yield-related traits (Tadesse et al., 2015).

In our previous study, the agronomically stable advanced lines were obtained from a cross between common wheat cultivar Chuannong16 (CN16 hereafter) and wild emmer accession D1 (Liu et al., 2019). We found that most of the tested advanced lines without *Gpc-B1* showed thousand kernel weight (TKW), GPC and grain mineral concentrations (e.g. GZnC and GFeC) simultaneous improvement (Wang, 2015). In this study, GWAS was used to analyze the genetic basis of GFeC, GZnC, and GMnC in a multi-parent population which consisted of wild emmer as backbone parent and its derived advanced lines. The objectives of the current study were to identify genomic regions associated with high GFeC, GZnC, and GMnC in a hexaploid wheat population, and to scan promising candidate genes using the whole genome assembly of wild emmer wheat (Avni et al., 2017) and Chinese Spring (Appels et al., 2018). The identified markers and genomic regions will provide essential information for cloning genes related to high GFeC, GZnC, and GMnC and be useful in biofortification breeding programs.

## Materials and Methods

### Plant Materials and Experimental Design

The same set of 161 advanced lines (Liu et al., 2019) derived from a cross between CN16 and D1 were used in the present study. Wheat plants were arranged in the field using a randomized complete block design with three replicates over two growing seasons (2015 and 2016) at the Chongzhou (2015CZ and 2016CZ) and Wenjiang (2015WJ and 2016WJ) experimental stations of Sichuan Agricultural University, China (Liu et al., 2019). Individual plants were spaced 10 cm apart within a 2 m row with 30 cm between rows. Each replicate contained 20 plants in a 2 m row. Cultivation and management followed local field production conditions. The soil types at fields of Wenjiang and Chongzhou experimental stations are paddy soil and yellow soil, respectively. Mature seeds were harvested from the middle six wheat plants of each row and measured for GFeC, GZnC, GMnC, and TKW.

### Phenotypic Measurements

Wheat grains were dried to a constant weight and then ground to a fine powder using Chopin CD1 AUTO (Renault, Boulogne-Billancourt, France). A 0.3 g powder samples were digested with a diacid mixture [HNO_3_ (4): HClO_4_ (1) (v/v)]. Atomic absorption Spectrometer (PinAAcle 900T, USA) was used to measure the GFeC, GZnC, and GMnC. The weight of 300 randomly sampled kernels (GB T 5519-2008, 2008) was recorded with an electronic balance to represent the TKW.

### Genotyping and Population Structure and Linkage Disequilibrium (LD) Analysis

The wheat genomic DNA was isolated from seedlings using a CTAB method (Murray and Thompson, 1980) with minor modification and then, genotyped using the DArT markers (https://www.diversityarrays.com/). The obtained data were filtered according to call rate (minimum threshold value of 85%) and reproducibility (minimum threshold value of 95%) (Liu et al., 2019). The recalled marker data with missing data >10% and minor allele frequency (MAF) <5% were further filtered (Liu et al., 2019). The retained 13,116 markers were used for further study. The linkage disequilibrium (LD) and population structure analyses were conducted as described by Liu et al. (2019).

### GWAS for GZnC, GFeC, and GMnC

The best linear unbiased prediction (BLUP) across four tested environments was performed using the META-R (Alvarado et al., 2015). The association analysis of markers and GZnC, GFeC, and GMnC was conducted using the general linear model (GLM) and mixed linear model (MLM) in TASSEL. The population structure (Q matrix) was used as a covariate to adjust population stratification. Kinship matrix (K) was calculated using the Scaled IBS method (Yu et al., 2006). A threshold *p-value* of –log_10_ (*p*) ≥ 3 (Alomari et al., 2018) was used to define significant marker-trait associations (MTAs). MTAs were displayed with a Manhattan plot using Haploview4.2 software (Barrett et al., 2004). Important *p-*value distributions were shown with a quantile-quantile plot. The prediction of candidate genes linked to MTAs was performed as described by Liu et al. (2019).

### Statistical Analysis

Analysis of variance (ANOVA) and estimates of heritability were performed using the META-R (Alvarado et al., 2015). *T*-test and the phenotypic Pearson correlation coefficients were calculated using SPSS version 22.0 (SPSS Inc., Chicago, IL, USA).

## Results

### Phenotypic Variation for GFeC, GZnC, and GMnC

**Figure 1:**
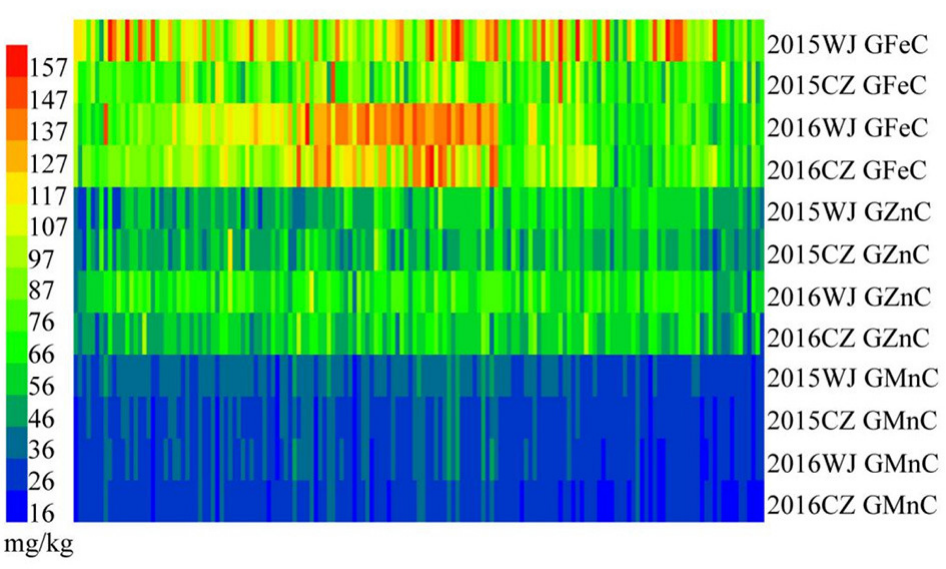
Heat map of GFeC, GZnC, and GMnC of the advanced lines grown at 2015WJ, 2015CZ, 2016WJ, and 2016CZ.

The variation for GFeC, GZnC, and GMnC in the advanced lines and their core parents were showed in Table 1. A wide range of phenotypic variation was found among the wheat advanced lines. These traits were significantly different among genotypes and showed medium-to-high heritability (0.67–0.98) (Table 1). Most genotypes displayed relatively stable GFeC, GZnC, and GMnC across four environments (2015WJ, 2015CZ, 2016WJ, and 2016CZ) (Figure 1).

**Table 1:** The variation for GFeC, GZnC, GMnC, and TKW in advanced lines under four environments.

**Traits**	**Env**.	**Parents**		**Advanced lines**			*****σ^2^ G*****	***H^**2**^***
		**CN16**	**D1**	**Mean ± SD**	**Range**	**CV**		
						**(%)**		
GFeC (mg/kg)	2015WJ	49.20^b^ ± 0.85	110.21^a^ ± 1.59	109.68^a^ ± 26.71	46.91–168.00	24.35	769.83	0.98
	2015CZ	38.15^c^ ± 0.65	115.47^a^ ± 0.87	83.71^b^ ± 19.03	49.54–152.27	22.73	392.65	0.97
	2016WJ	47.51^b^ ± 0.55	92.69^a^ ± 0.96	102.27^a^ ± 29.79	52.94–160.51	29.13	992.57	0.98
	2016CZ	44.19^b^ ± 0.98	98.41^a^ ± 1.74	97.65^a^ ± 23.83	42.44–157.01	24.4	659.20	0.97
GZnC (mg/kg)	2015WJ	40.60^c^ ± 0.43	85.98^a^ ± 0.28	57.48^b^ ± 10.76	31.15–103.92	18.72	311.24	0.97
	2015CZ	41.64^b^ ± 0.20	80.91^a^ ± 1.17	55.99^b^ ± 12.12	30.17–120.52	21.65	331.89	0.98
	2016WJ	37.07^c^ ± 1.25	97.87^a^ ± 0.16	68.75^b^ ± 12.02	31.67–111.28	17.48	269.20	0.96
	2016CZ	36.67^c^ ± 0.83	81.62^a^ ± 0.87	60.24^b^ ± 11.61	32.09–100.71	19.27	206.58	0.96
GMnC (mg/kg)	2015WJ	25.68^b^ ± 1.11	35.42^a^ ± 0.62	37.14^a^ ± 5.50	21.69–53.85	14.81	19.47	0.94
	2015CZ	23.43^b^ ± 0.76	33.42^a^ ± 0.84	33.37^a^ ± 4.98	21.94–48.59	14.92	14.69	0.93
	2016WJ	27.03^a^ ± 0.38	33.54^a^ ± 0.46	32.81^a^ ± 5.83	16.28–51.35	17.77	34.77	0.95
	2016CZ	25.57^a^ ± 0.81	31.22^a^ ± 0.96	30.36^a^ ± 4.81	20.39–45.34	15.84	18.41	0.95
TKW (g)	2015WJ	43.19^a^ ± 4.99	19.21^b^ ± 4.39	46.08^a^ ± 4.64	30.70–57.00	10.07	18.58	0.73
	2015CZ	49.33^a^ ± 1.54	15.00^b^ ± 0.67	52.90^a^ ± 4.35	37.08–64.63	8.22	24.49	0.89
	2016WJ	49.22^a^ ± 1.84	32.80^b^ ± 1.61	49.88^a^ ± 4.28	33.93–60.11	8.58	17.21	0.88
	2016CZ	42.70^b^ ± 4.97	31.63^c^ ± 0.71	49.06^a^ ± 4.52	35.00–60.88	9.21	14.92	0.67

a, b, c *Significant differences at the 0.05 level. CV, coefficient of variation; Env, environment; σ^2^ G, genotypic variance; H^2^, heritability*.

The wild emmer D1 had significantly (*P* < 0.05) higher GFeC (mean range 92.69–115.47 mg/kg) compared to that of CN16 (mean range 38.15–49.20 mg/kg) across all test environments (Table 1). The GFeC of the advanced lines were ranged from 42.44 to 168.00 mg/kg (mean range 83.71–109.68 mg/kg) across four environments. The lowest GFeC (42.44 mg/kg) was recorded at 2016CZ, whereas the highest GFeC (168.00 mg/kg) was recorded at 2015 WJ.

The D1 showed significantly (*P* < 0.05) higher GZnC (mean range 80.91–97.87 mg/kg) compared to that of CN16 (mean range 36.67–41.64 mg/kg) across four environments (Table 1). The GZnC of the advanced lines ranged from 55.99 to 68.75 mg/kg across environments. The lowest (30.17 mg/kg) and highest GZnC (120.52 mg/kg) were recorded at 2015CZ.

There is no significant difference in GMnC between D1 and CN16, except in two environments (2015CZ and 2015WJ). The GMnC of the advanced lines were ranged from 16.28 to 53.85 mg/kg (mean range 30.36–37.14 mg/kg) across four environments. The lowest GMnC (16.28 mg/kg) was recorded in 2016 at Wenjiang, whereas the highest GMnC (53.85 mg/kg) was recorded in 2015 at Wenjiang (Table 1).

The mean TKW of the advanced lines under 2015WJ, 2015CZ, 2016WJ, and 2016CZ environments was 46.08, 52.90, 49.88, and 49.06 g, respectively, which were all higher than those of female parent CN16 (Table 1).

### Phenotypic Frequency Distributions and Pearson's Correlation Analysis

**Figure 2:**
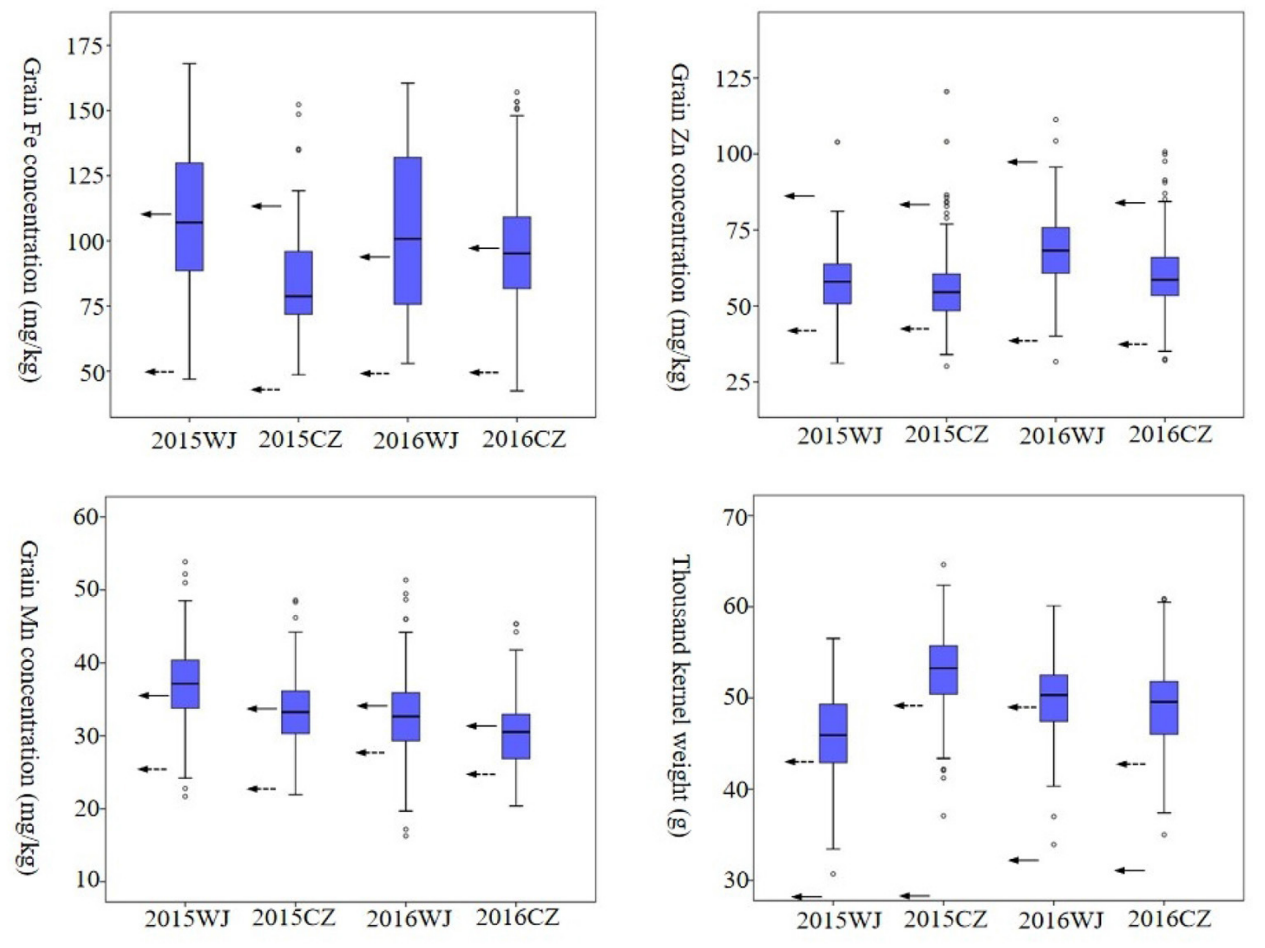
Frequency distribution of GFeC, GZnC, GMnC, and TKW under four environments in the advanced lines. Solid arrows represent the values of D1. Dashed arrows represent the values of CN16.

Frequency distributions of advanced lines for GFeC, GZnC, and GMnC with an indication of the bi-parents controls were presented in Figure 2. The advanced lines exhibited normal distributions for all micronutrient variables under four environments. The highest frequency distribution of GFeC showed a large difference in four environments, implying this variable was subjected to environmental factors. In contrast, the highest frequency distributions of GZnC, GMnC, and TKW were clustered in a narrow range of 50–70 mg/kg, 30–40 mg/kg, and 45–55 g, respectively (Figure 2). Most wheat lines had both higher grain micronutrient concentrations and TKW than those of CN16 in four tested environments (Supplementary Table 1).

Pearson's correlation analysis revealed highly significant positive correlations among the GFeC, GZnC, and GMnC base on BLUP. The TKW had a significant positive correlation (*P* < 0.05) with GMnC and no negative correlation with GFeC and GZnC (Table 2).

**Table 2:** Pearson's correlation coefficients of GFeC, GZnC, GMnC, and TKW in advanced lines based on BLUP data.

**Correlations**	**GFeC**	**GZnC**	**GMnC**
GZnC	0.267^**^		
GMnC	0.395^**^	0.323^**^	
TKW	0.056	0.006	0.175^*^

* *and*

** *,significant at the 0.05 and 0.01 level (2-tailed), respectively*.

### Population Structure and Linkage Disequilibrium (LD) Analysis

Based on population structure and LD analysis, the 161 RILs could be divided into three subgroups and the LD decay distances for the A, B, and D subgenomes and the entire genome were about 9, 12, 13, and 12 cM, respectively, which were rapidly decreased with increasing pairwise distance as described previously (Liu et al., 2019).

### GWAS for GFeC, GZnC, and GMnC in Advanced Lines

**Figure 3:**
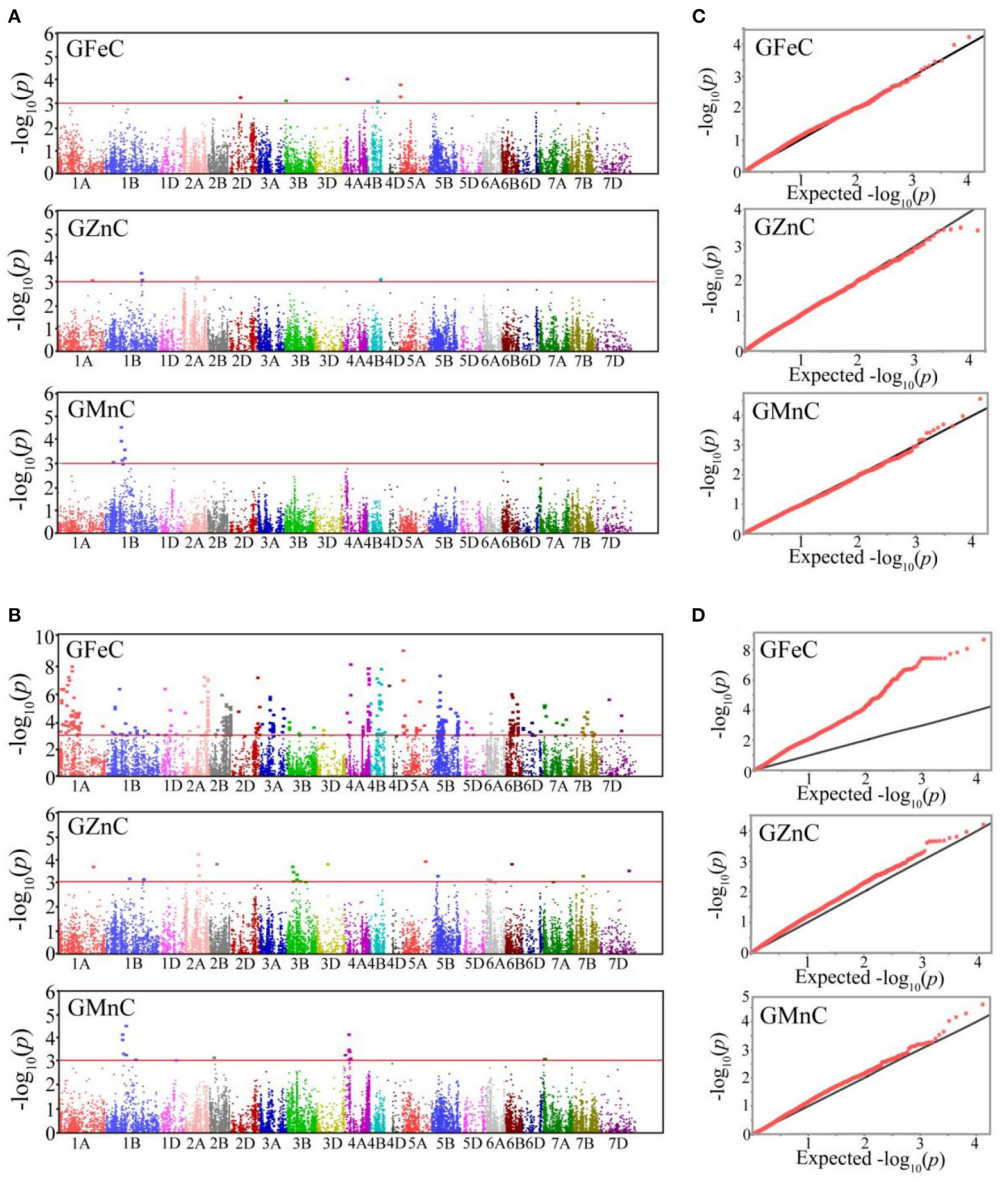
Manhattan plots of GWAS scan for GFeC, GZnC, and GMnC in four environments. Significant MTAs identified by MLM **(A)** and GLM **(B)**. Red lines: the –log_10_(*p*) threshold of 3.00. The quantile-quantile charts of MLM **(C)** and GLM **(D)**. Black line: the expected values.

**Figure 4:**
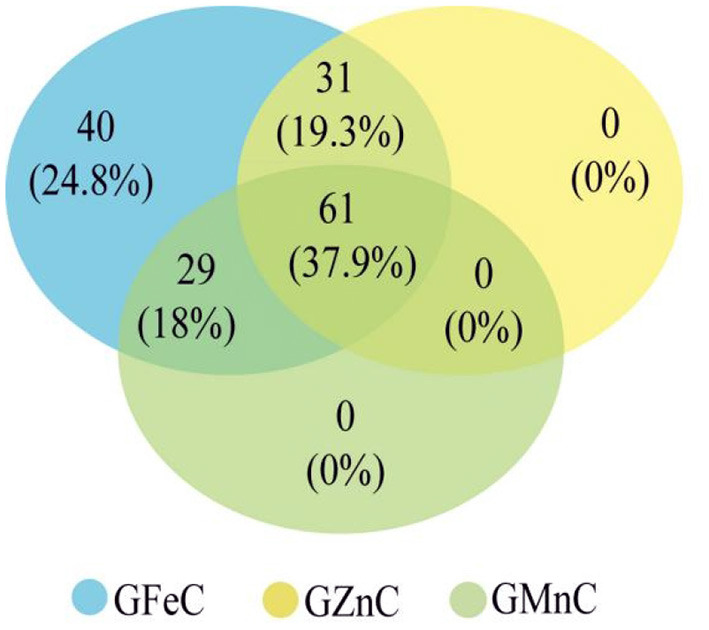
Potential genotypes with high micronutrient concentrations selected from the RILs based on high-confidence MTAs associated with GFeC, GZnC, and GMnC.

Based on GLM, 556 MTAs relative to grain micronutrient concentrations were distributed on all wheat chromosomes with phenotypic variance explained (PVE) of 5.45–18.70% (Table 3). In the MLM, 22 MTAs were detected with PVEs of 7.18–11.84% (Table 3). The Manhattan and quantile-quantile charts based on the GWAS were presented in Figure 3. The GLM and MLM models as well as quantile-quantile charts were considered together to remove the potential false-positive associations. Fourteen high-confidence MTAs were detected by both GLM and MLM (Table 4). Of these, six MTAs relative to GFeC were found on chromosomes 3B, 4A, 4B, 5A, and 7B (Table 4). Three MTAs for GZnC were clustered on chromosomes 1A and 2A (Table 4). Five MTAs for GMnC were on chromosome 1B (Table 4). According to the results of GWAS, the top-ranking 61 (37.9%) genotypes with a high number of MTAs that associated with GFeC, GZnC, and GMnC (Figure 4, Supplementary Table 2) were identified. A wide range of phenotypic variation for GFeC (range 62.73–125.58 mg/kg), GZnC (range 47.00–75.09 mg/kg), GMnC (range 21.71–49.50 mg/kg), and TKW (range 42.64–53.87 g) was observed among these genotypes (Supplementary Table 2). Moreover, *t*-test showed that the average values of GFeC (100.62 mg/kg), GZnC (60.74 mg/kg), GMnC (34.56 mg/k), and TKW (49.54 g) for the 61 genotypes were significantly higher than those of the parent CN16 (*p* < 0.001) (Supplementary Table 2).

**Table 3:** Significant MTAs identified by GWAS using GLM and MLM model.

**Trait**	**Model**	**MTA^**a**^**	**Average –log_**10**_(*p*)**	**Range –log_**10**_(*p*)**	**Average PVE (%)**	**Range PVE (%)**	**No. Shared^**b**^**
GFeC	MLM	7	3.40	3.05–4.06	8.57	7.18–10.45	6
	GLM	512	4.05	3.00–8.93	8.44	5.83–18.70	
GZnC	MLM	7	3.19	3.10–3.41	7.68	7.30–8.16	3
	GLM	24	3.38	3.02–4.19	7.91	6.80–9.92	
GMnC	MLM	8	3.46	3.01–4.56	8.81	7.18–11.84	5
	GLM	20	3.39	3.04–4.48	6.35	5.45–8.43	

a *The number of MTAs identified by MLM or GLM*.

b *The number of shared significant MTAs*.

*PVE, phenotypic variation explained*.

**Table 4:** Significant high-confidence MTAs for GFeC, GZnC, and GMnC identified by MLM and GLM.

**Trait**	**MTA**	**Chromosome**	**Position (cM)**	**MLM**		**GLM**	
				**–log_**10**_(*p*)**	**PVE (%)**	**–log_**10**_(*p*)**	**PVE (%)**
GFeC	1210301	3B	10.84	3.16	8.10	3.94	8.44
	2255722	4A	46.59	4.06	10.45	7.99	17.12
	3024845	4B	108.47	3.11	8.31	7.63	17.42
	2258533	5A	0.00	3.31	7.85	8.93	18.70
	3033960	5A	0.38	3.82	9.47	6.87	14.81
	1274451	7B	94.72	3.05	7.18	3.56	7.49
GZnC	1136167	1A	359.46	3.10	7.60	3.69	8.69
	1077698	2A	139.90	3.24	7.88	4.19	9.92
	1234362	2A	145.15	3.19	7.86	3.32	7.70
GMnC	3023738	1B	167.84	3.97	11.04	3.91	7.53
	2326413	1B	170.19	4.56	11.84	4.11	7.81
	1114828	1B	181.69	3.17	8.16	3.33	6.57
	1215559	1B	204.13	3.27	8.04	4.48	8.43
	1105781	1B	211.25	3.61	8.98	3.27	6.15

*PVE, phenotypic variation explained*.

*Chromosomal location information based on the wheat consensus map version 3.0 (http://www.diversityarrays.com/sequence-maps)*.

### Candidate Genes That May Be Associated With GFeC, GZnC, and GMnC

The high-confidence MTAs were selected to predicate candidate genes using the annotated Chinese Spring genome sequence (RefSeq v.1.0) and wild emmer genome sequence (WEWSeq v.1.0). A total of 38 putative candidate genes for micronutrient concentrations were predicted (Supplementary Table 3). These genes can be roughly classified into four groups based on the types of proteins they encoded. The first group of candidates encoded enzymes related to iron-dependent dioxygenase, metal-dependent hydrolase, phosphatase, and methyltransferases. The second group consisted of transporter proteins, such as sugar transporter, ABC transporter, and heavy metal transport proteins. The third group included MYB transcription factor, zinc finger family, and transmembrane proteins. The last group consisted of proteins related to plant defense responses, such as receptor-like kinase, leucine-rich repeat-containing protein, and mitogen-activated kinase (MAPK) (Supplementary Table 3).

## Discussion

The poor bioavailability and low concentration of grain micronutrients (especially Zn and Fe) in cultivated wheat are common problems (Xu et al., 2012). Enhancement of the wheat grain nutritional value through genetic biofortification is available (Borrill et al., 2014). The wild emmer accessions are a valuable resource for micronutrient improvement in wheat (Chatzav et al., 2010). In the present study, the same set of 161 advanced lines derived from wild emmer (Liu et al., 2019) were phenotyped in four environments and genotyped using the DArT markers to understand the genetic basis of GFeC, GZnC, and GMnC accumulation in wheat. GWAS identified 14 high-confidence MTAs for GFeC, GZnC, and GMnC by both GLM and MLM (Table 4), and 38 putative candidate genes were predicate using the wheat sequences annotation (RefSeq v.1.0; WEWSeq v.1.0).

We have found that the GFeC, GZnC and GMnC of advanced lines derived from wild emmer were significantly higher than those of CN16, with the exception of GMnC in 2016WJ and 2016CZ. Medium-to-high heritability (0.67–0.98) were estimated for GFeC, GZnC, GMnC, and TKW, which indicated these traits were largely governed by genetic factors. Similar heritability for these traits has been reported in previous studies (Velu et al., 2017; Bhatta et al., 2018). These results demonstrated the potential value of wild emmer for grain micronutrient improvement in wheat. On the other hand, we have observed significant positive correlations among GFeC, GZnC, and GMnC and no significant negative correlations between grain micronutrient concentrations and TKW. Our results were consistent with a previous study that the wild emmer GZnC and GFeC were positively correlated (Peleg et al., 2009). These results indicate that the alleles for GZnC and GFeC are either co-segregated or have a pleiotropic effect.

Six MTAs on chromosomes 3B, 4A, 4B, 5A (2), and 7B are related to GFeC. Two MTAs (2258533 and 3033960) at position 0–0.38 cM on chromosome 5A are located in the same GFeC-QTL region as reported by Peleg et al. (2009). The MTA 2255722 (46.59 cM) was located in a GFeC-QTL region between markers *wmc468*-*barc170* at position 32.6–50.8 cM on chromosome 4A (Pu et al., 2014). The remaining 3 MTAs on 3B, 4B, and 7B in our studies might be novel.

Among the three MTAs related to high GZnC, two MTAs (1077698 and 1234362) on chromosome 2A at position 139.90–145.15 cM were located in the same interval of wild emmer GZnC-QTL (112.4 ± 35.0 cM) (Peleg et al., 2009). The position of MTA 1136167 (359.46 cM) in the current study is different to the GZnC-QTL (39 cM) on chromosome 1A (Roshanzamir et al., 2013).

The five MATs related to high GMnC located on chromosome 1B. Several GMnC-QTLs have been reported on different wheat chromosomes such as 1A, 2A, 2B, 2D, 3A, 3B, 4B, 4D, 5A, 5D, 6B, and 7B (Shi et al., 2013; Pu et al., 2014; Bhatta et al., 2018). To our best knowledge, there have no reports of GMnC QTLs on chromosome 1B. Noteworthy, we have found that some wheat genotypes carrying MTAs for high GFeC, GZnC, and GMnC, but without reduction in TKW (Figure 4, Supplementary Table 2). The GFeC, GZnC, GMnC, and TKW of these wheat genotypes were significantly higher than those of the parent CN16 (*p* < 0.001) (Supplementary Table 2). These results indicate the presence of novel wild emmer alleles that would be used for grain minerals biofortification.

In the present study, the high-confidence MTAs were used to predicate putative candidate genes. We found that MYB transcription factor, dioxygenase, and hydrolase genes may play important roles in conferring high GFeC. For example, the dioxygenase genes in higher plants were reported to be involved in the absorption and transport of Fe (Kobayashi and Nishizawa, 2012). The MYB transcription factor in *Malus xiaojinensis* was responded to Fe deficiency stress (Han et al., 2020). In addition, we have identified hydrolase family protein/HAD-superfamily protein, thioredoxin-like protein AAED1, kelch repeat-containing protein, receptor-like protein kinase, and HD domain-containing metal-dependent phosphohydrolase family protein on chromosome 4A and 3B. The previous study showed that the kelch repeat-containing protein was associated with Fe acquisition and metal homeostasis (Kawahara et al., 2017).

Our results indicated that zinc ion binding (FAR1), heavy metal transport, zinc finger (C3HC4-type RING finger) family, and mitogen-activated protein kinase (MAPK) may be promising candidates for GZnC (Supplementary Table 3). Previous studies found that the metal transporter (*ZIP1*) could transport Zn in wild emmer wheat (Durmaz et al., 2011). A recent study reported that a MAPK-related gene may be involved in Zn accumulation in chickpea seeds (Upadhyaya et al., 2016; Alomari et al., 2018).

We have identified S-adenosyl-L-methionine-dependent methyltransferases, ABC transporter, phosphatase, and argininosuccinate synthase as the candidates for GMnC. For example, the ABC transporter was proved to be involved in the uptake and transport of manganese ions (Li and Tan, 2012). Arginase is a type of manganese enzyme, and therefore argininosuccinate synthase can lead to Mn accumulation (Li and Tan, 2012).

Previous studies have demonstrated that the grain yield and micronutrients (Fe, Zn, and Mn) are negatively correlated in wheat, making the simultaneous improvement of these two traits challenging (Shi et al., 2013; Bhatta et al., 2018). However, Peleg et al. (2008) reported neither negative nor positive associations between grain micronutrient concentrations and yield in wild emmer wheat. In our previous studies, we have introgressed the high GFeC, GZnC, and TKW traits of wild emmer into common wheat through wide hybridization (Wu et al., 2008; Wang, 2015). In this study, we found no significant negative correlation between grain micronutrient concentrations (GFeC, GZnC, and GMnC) and TKW in the advanced lines. Our findings demonstrate the improvement of grain micronutrient concentrations (GFeC, GZnC, and GMnC) without sacrificing grain yield could be possible by the exploitation of wild emmer MTAs.

## Conclusion

Our results show the importance of wild emmer as valuable germplasm for the improvement of grain micronutrient concentrations in wheat. The advanced lines derived from wild emmer showed high grain micronutrients and a weak correlation between grain micronutrients and TKW, indicating simultaneous improvement of grain micronutrient concentrations (especially GFeC and GZnC) and TKW could be possible. The top-ranking 61 wheat genotypes carrying MTAs for high GFeC, GZnC, and GMnC without reduction in TKW could be valuable donor parents for wheat biofortification breeding.

A GWAS identified 14 high-confidence significant MTAs that were associated with GFeC, GZnC, and GMnC, of which nine MTAs were novel. In total, 38 putative candidate genes for these MTAs were predicated. The MTAs and associated candidate genes provide information for fine mapping and cloning of genes related to GFeC, GZnC, and GMnC and for wheat biofortification breeding programs.

## Data Availability Statement

The original contributions presented in the study are included in the article/Supplementary Material, further inquiries can be directed to the corresponding author.

## Author Contributions

BW, JL, and LH designed the experiment. JL, TL, and GT performed experiments. JL analyzed data and drafted the manuscript. YL and ZY provided the genotyping data. YZ, DL, and BW provided their valuable suggestions. LH and BW revised the manuscript. All authors read and approved the final manuscript.

## Conflict of Interest

The authors declare that the research was conducted in the absence of any commercial or financial relationships that could be construed as a potential conflict of interest. The reviewer VG declared a past collaboration with two of the authors JL and BW to the handling editor.
