# Supplementary material for: Genome-Wide Association Study for Grain Micronutrient Concentrations in Wheat Advanced Lines Derived From Wild Emmer
Source: Front Plant Sci. 2021 May 14;12:651283. doi: 10.3389/fpls.2021.651283 (PMC8160437; doi:10.3389/fpls.2021.651283)
Supplement: Supplementary file 1 [file Data_Sheet_1.docx]

Supplementary Material

**Genome-wide association study for grain micronutrient concentrations in wheat advanced lines derived from wild emmer**

Jia Liu^1,2†^, Lin Huang^2†^, Tingxuan Li^4^, Yaxi Liu^1,2,3^, Zehong Yan^1,2,3^, Guan Tang^2^, Youliang Zheng^1,2,3^, Dengcai Liu^1,2,3^, Bihua Wu^1,2,3^****** Correspondence: Bihua Wu: wubihua2017@126.com

**Supplementary Tables**

**Supplementary Table 1.** List of materials with GFeC, GZnC, GMnC and TKW higher than the parent CN16 in the four environments.

| Environment | Accession | GFeC | GZnC | GMnC | TKW |
| --- | --- | --- | --- | --- | --- |
|  |  | mg/kg | mg/kg | mg/kg | g |
| 2015WJ | CN16 | 49.20 | 40.61 | 25.68 | 43.19 |
| 2015WJ | BAd160-6 | 78.38 | 45.04 | 35.70 | 51.36 |
| 2015WJ | BAd95-2 | 110.60 | 53.66 | 38.04 | 44.20 |
| 2015WJ | BAd70-2 | 88.54 | 78.75 | 34.80 | 53.09 |
| 2015WJ | BAd71-2 | 84.03 | 49.01 | 34.60 | 52.29 |
| 2015WJ | BAd128-2 | 91.10 | 48.12 | 41.64 | 50.25 |
| 2015WJ | BAd152-7 | 105.96 | 52.94 | 44.74 | 44.91 |
| 2015WJ | BAd99-5 | 78.02 | 52.13 | 30.32 | 46.27 |
| 2015WJ | BAd162-5 | 122.28 | 77.07 | 38.76 | 51.71 |
| 2015WJ | BAd182-5 | 115.48 | 73.54 | 39.59 | 45.79 |
| 2015WJ | BAd122-3 | 86.13 | 59.23 | 38.74 | 47.34 |
| 2015WJ | BAd169-4 | 135.79 | 74.05 | 38.67 | 43.50 |
| 2015WJ | BAd104-2 | 142.99 | 63.86 | 39.97 | 53.86 |
| 2015WJ | BAd164-5 | 99.91 | 45.29 | 34.10 | 54.04 |
| 2015WJ | BAd76-5 | 159.86 | 72.33 | 37.31 | 45.18 |
| 2015WJ | BAd129-4 | 52.29 | 67.01 | 37.31 | 49.99 |
| 2015WJ | BAd124-4 | 87.67 | 103.92 | 39.21 | 43.21 |
| 2015WJ | BAd122-5 | 135.64 | 57.31 | 43.40 | 43.70 |
| 2015WJ | BAd137-5 | 152.06 | 63.27 | 43.96 | 49.30 |
| 2015WJ | BAd180-3 | 157.32 | 64.76 | 53.85 | 48.11 |
| 2015WJ | BAd89-1 | 93.44 | 62.82 | 38.93 | 44.48 |
| 2015WJ | BAd179-4 | 91.43 | 65.29 | 34.72 | 48.14 |
| 2015WJ | BAd129-2 | 126.13 | 58.08 | 39.23 | 50.23 |
| 2015WJ | BZn128-5 | 120.97 | 76.14 | 52.17 | 50.69 |
| 2015WJ | BAd183-3 | 134.25 | 44.07 | 48.52 | 51.24 |
| 2015WJ | BFe164-6 | 151.77 | 76.96 | 42.15 | 53.57 |
| 2015WJ | AdA95-5 | 95.92 | 67.73 | 35.89 | 43.83 |
| 2015WJ | AdA95-4 | 159.47 | 73.16 | 33.57 | 44.90 |
| 2015WJ | AdA95-1 | 103.17 | 71.02 | 42.30 | 46.11 |
| 2015WJ | AdA168-(1) | 160.74 | 60.43 | 38.85 | 49.20 |
| 2015WJ | AdA168-(16) | 114.12 | 46.71 | 30.74 | 43.29 |
| 2015WJ | AdA168-(20) | 90.59 | 73.99 | 35.00 | 47.52 |
| 2015WJ | AdA168-1 | 85.52 | 69.47 | 35.42 | 47.94 |
| 2015WJ | AdA168-4 | 141.19 | 65.29 | 37.08 | 52.59 |
| 2015WJ | AdA73-8 | 70.95 | 64.86 | 32.84 | 47.67 |
| 2015WJ | AdA73-2 | 110.57 | 59.81 | 30.50 | 46.02 |
| 2015WJ | AdA73-3 | 156.08 | 64.44 | 32.37 | 52.59 |
| 2015WJ | AdA73-6 | 84.14 | 54.58 | 34.80 | 45.80 |
| 2015WJ | AdA73-10 | 87.73 | 59.43 | 35.23 | 45.38 |
| 2015WJ | AdA168-9 | 63.76 | 52.15 | 36.85 | 47.02 |
| 2015WJ | AdA168-5 | 133.30 | 63.11 | 33.21 | 48.19 |
| 2015WJ | AdA168-6 | 107.07 | 69.50 | 33.82 | 50.20 |
| 2015WJ | AdA73-9 | 71.99 | 69.85 | 44.34 | 47.56 |
| 2015WJ | BAd64-5 | 102.57 | 49.20 | 38.28 | 43.34 |
| 2015WJ | BAd63-4 | 90.23 | 70.67 | 37.09 | 50.59 |
| 2015WJ | AdA73-1 | 74.23 | 60.46 | 28.42 | 46.60 |
| 2015WJ | AdA168-2 | 82.99 | 61.69 | 32.98 | 57.00 |
| 2015WJ | BAd178-6 | 168.00 | 63.80 | 41.75 | 47.66 |
| 2015WJ | AdA168-(6) | 151.80 | 74.59 | 38.43 | 47.97 |
| 2015WJ | AdA168-(17) | 104.33 | 61.00 | 30.03 | 46.77 |
| 2015WJ | AdA168-(10) | 107.10 | 61.54 | 30.36 | 44.54 |
| 2015WJ | AdA168-(3) | 159.66 | 60.91 | 35.03 | 47.12 |
| 2015WJ | AdA168-(14) | 145.59 | 64.69 | 34.76 | 44.72 |
| 2015WJ | AdA168-(4) | 154.18 | 60.96 | 34.43 | 47.78 |
| 2015WJ | BAd168-3 | 129.89 | 62.03 | 37.97 | 43.67 |
| 2015WJ | AdA168-(5) | 153.71 | 60.76 | 33.09 | 52.35 |
| 2015WJ | AdA168-(2) | 136.70 | 62.91 | 32.29 | 46.22 |
| 2015WJ | AdA168-(21) | 99.56 | 65.35 | 33.82 | 46.27 |
| 2015WJ | AdA168-(11) | 88.85 | 60.84 | 38.32 | 51.53 |
| 2015WJ | AdA168-(18) | 104.10 | 72.76 | 38.83 | 50.58 |
| 2015WJ | AdA168-(8) | 89.30 | 48.85 | 28.42 | 47.69 |
| 2015WJ | AdA168-(12) | 101.32 | 53.91 | 27.64 | 50.21 |
| 2015WJ | AdA168-(19) | 109.04 | 65.97 | 33.78 | 45.15 |
| 2015WJ | AdA168-(7) | 153.10 | 46.00 | 28.32 | 50.21 |
| 2015WJ | AdA73-(3) | 71.97 | 49.50 | 35.66 | 52.54 |
| 2015WJ | AdA73-(4) | 71.70 | 49.51 | 33.31 | 47.43 |
| 2015WJ | AdA73-(2) | 77.21 | 49.47 | 32.41 | 51.13 |
| 2015WJ | AdA116-(5) | 59.57 | 52.37 | 26.87 | 47.80 |
| 2015WJ | AdA116-⑩ | 76.62 | 58.91 | 33.39 | 49.32 |
| 2015WJ | AdA116-(6) | 100.95 | 52.17 | 35.49 | 47.83 |
| 2015WJ | AdA180-2 | 84.99 | 55.04 | 33.80 | 44.26 |
| 2015WJ | BAd127-1 | 153.84 | 60.72 | 37.82 | 49.85 |
| 2015WJ | BAd141-6 | 106.48 | 60.87 | 37.16 | 43.93 |
| 2015WJ | BAd123-2 | 81.64 | 55.86 | 35.35 | 49.47 |
| 2015WJ | BAd70-4 | 113.25 | 45.31 | 36.89 | 53.80 |
| 2015WJ | BAd161-5 | 132.64 | 54.71 | 38.87 | 46.84 |
| 2015WJ | BAd177-6 | 90.21 | 45.34 | 41.67 | 50.04 |
| 2015WJ | BAd117-3 | 84.87 | 60.09 | 40.73 | 48.57 |
| 2015WJ | BAd133-5 | 117.00 | 63.38 | 34.66 | 52.67 |
| 2015WJ | BAd181-1 | 110.28 | 45.36 | 42.79 | 45.83 |
| 2015WJ | BAd156-2 | 118.87 | 62.63 | 35.67 | 51.42 |
| 2015WJ | BAd185-1 | 102.70 | 54.41 | 44.77 | 43.31 |
| 2015WJ | BAd180-5 | 94.68 | 47.66 | 38.46 | 53.02 |
| 2015WJ | BAd95-4 | 104.53 | 52.02 | 38.22 | 45.85 |
| 2015WJ | BAd137-4 | 125.00 | 53.27 | 37.97 | 49.26 |
| 2015WJ | BAd170-6 | 86.09 | 57.44 | 37.53 | 43.40 |
| 2015WJ | BAd97-2 | 148.75 | 56.05 | 40.38 | 52.07 |
| 2015WJ | BAd134-3 | 103.65 | 51.70 | 34.35 | 44.87 |
| 2015WJ | BAd84-5 | 116.80 | 53.19 | 40.65 | 45.67 |
| 2015WJ | BAd184-1 | 128.25 | 49.78 | 31.51 | 51.12 |
| 2015WJ | BAd107-4 | 129.13 | 55.53 | 36.27 | 43.25 |
| 2015WJ | BAd142-1 | 113.08 | 52.74 | 40.12 | 46.45 |
| 2015WJ | BAd145-3 | 90.89 | 58.77 | 41.95 | 47.56 |
| 2015WJ | BAd100-3 | 144.41 | 53.83 | 32.88 | 54.37 |
| 2015WJ | BAd163-2 | 107.83 | 45.50 | 48.49 | 56.53 |
| 2015WJ | BAd124-6 | 82.98 | 43.29 | 40.39 | 49.35 |
| 2015WJ | BAd106-1 | 80.58 | 52.07 | 37.30 | 45.34 |
| 2015WJ | BZn130-3 | 133.88 | 52.25 | 38.96 | 48.95 |
| 2015WJ | BAd159-3 | 79.64 | 66.37 | 31.81 | 43.42 |
| 2015WJ | BAd119-4 | 114.00 | 58.93 | 30.68 | 47.04 |
| 2015WJ | BAd183-4 | 85.91 | 57.95 | 41.96 | 43.82 |
| 2015WJ | BAd173-5 | 87.04 | 45.45 | 42.56 | 44.95 |
| 2015WJ | BAd175-7 | 89.38 | 49.20 | 44.05 | 46.39 |
| 2015WJ | BAd111-1 | 79.04 | 54.88 | 43.93 | 50.89 |
| 2015WJ | BAd174-1 | 141.29 | 49.60 | 30.09 | 45.52 |
| 2015WJ | BAd178-2 | 102.50 | 64.14 | 42.95 | 45.26 |
| 2015WJ | BAd170-2 | 91.64 | 51.46 | 47.70 | 47.33 |
| 2015WJ | BAd109-2 | 101.97 | 50.41 | 44.57 | 53.59 |
| 2015WJ | BAd157-4 | 138.02 | 61.24 | 39.09 | 54.33 |
| 2015CZ | CN16 | 38.15 | 41.64 | 23.43 | 49.33 |
| 2015CZ | BAd170-6 | 79.06 | 59.09 | 36.74 | 53.90 |
| 2015CZ | BAd160-6 | 65.85 | 45.34 | 32.34 | 55.74 |
| 2015CZ | BAd129-4 | 76.06 | 82.80 | 35.14 | 55.97 |
| 2015CZ | BAd107-1 | 83.37 | 67.31 | 35.01 | 55.35 |
| 2015CZ | BAd143-3 | 74.19 | 43.27 | 29.58 | 52.38 |
| 2015CZ | BAd71-3 | 91.81 | 56.43 | 35.26 | 58.02 |
| 2015CZ | BAd64-5 | 78.47 | 53.69 | 36.84 | 51.61 |
| 2015CZ | BAd168-3 | 86.74 | 58.45 | 33.26 | 50.15 |
| 2015CZ | BAd167-6 | 75.36 | 65.07 | 38.35 | 53.20 |
| 2015CZ | BAd127-1 | 71.81 | 51.51 | 32.26 | 54.91 |
| 2015CZ | BAd177-2 | 77.18 | 57.44 | 34.63 | 56.07 |
| 2015CZ | BAd123-3 | 101.58 | 50.73 | 24.32 | 58.23 |
| 2015CZ | BAd123-2 | 71.98 | 55.47 | 31.36 | 57.24 |
| 2015CZ | BAd70-4 | 72.72 | 48.88 | 31.45 | 59.44 |
| 2015CZ | BAd161-5 | 80.90 | 42.43 | 36.38 | 56.55 |
| 2015CZ | BAd177-6 | 82.51 | 49.86 | 39.82 | 53.64 |
| 2015CZ | BAd117-3 | 62.07 | 51.49 | 35.65 | 53.34 |
| 2015CZ | BAd133-5 | 134.78 | 67.23 | 30.73 | 57.14 |
| 2015CZ | BAd181-1 | 103.81 | 48.30 | 39.54 | 53.38 |
| 2015CZ | BAd156-2 | 81.33 | 56.44 | 32.81 | 54.06 |
| 2015CZ | BAd185-1 | 64.92 | 46.12 | 40.21 | 52.21 |
| 2015CZ | BAd153-3 | 72.59 | 45.25 | 35.67 | 58.29 |
| 2015CZ | BAd180-5 | 90.68 | 53.98 | 35.27 | 57.18 |
| 2015CZ | BAd176-4 | 64.32 | 58.75 | 39.48 | 52.22 |
| 2015CZ | BAd174-6 | 119.11 | 59.27 | 23.73 | 50.44 |
| 2015CZ | BAd139-6 | 107.16 | 46.54 | 40.56 | 52.70 |
| 2015CZ | BAd175-6 | 76.30 | 59.24 | 34.06 | 55.70 |
| 2015CZ | BAd146-4 | 66.66 | 120.52 | 38.56 | 62.24 |
| 2015CZ | BAd95-4 | 78.17 | 54.52 | 34.95 | 51.78 |
| 2015CZ | BAd170-1 | 57.80 | 50.08 | 36.82 | 57.50 |
| 2015CZ | BAd137-4 | 90.83 | 85.75 | 33.25 | 56.64 |
| 2015CZ | BAd97-2 | 71.21 | 49.91 | 35.43 | 56.86 |
| 2015CZ | BAd134-3 | 76.50 | 47.71 | 30.67 | 53.46 |
| 2015CZ | BAd144-1 | 95.83 | 51.20 | 32.82 | 57.32 |
| 2015CZ | BAd84-5 | 98.30 | 52.54 | 38.39 | 50.45 |
| 2015CZ | BAd184-1 | 98.46 | 55.19 | 26.27 | 54.65 |
| 2015CZ | BAd107-4 | 64.92 | 63.88 | 32.16 | 52.06 |
| 2015CZ | BAd128-6 | 75.07 | 54.52 | 24.06 | 55.85 |
| 2015CZ | BAd145-3 | 86.50 | 68.60 | 36.39 | 53.20 |
| 2015CZ | BAd163-2 | 81.86 | 44.39 | 42.36 | 52.94 |
| 2015CZ | BAd124-6 | 101.44 | 61.35 | 35.46 | 54.06 |
| 2015CZ | BAd173-5 | 78.58 | 49.00 | 39.67 | 57.68 |
| 2015CZ | BAd175-7 | 135.23 | 84.37 | 38.47 | 51.66 |
| 2015CZ | BAd111-1 | 85.53 | 59.67 | 39.83 | 60.33 |
| 2015CZ | BAd174-1 | 98.47 | 67.42 | 25.16 | 52.59 |
| 2015CZ | BAd178-2 | 103.49 | 62.60 | 38.35 | 49.90 |
| 2015CZ | BAd170-2 | 48.63 | 46.29 | 42.42 | 57.96 |
| 2015CZ | BAd109-2 | 148.56 | 57.29 | 38.44 | 62.36 |
| 2015CZ | BAd157-4 | 73.41 | 51.24 | 34.37 | 56.78 |
| 2015CZ | BAd95-2 | 66.06 | 45.93 | 32.73 | 55.08 |
| 2015CZ | BAd70-2 | 69.21 | 55.80 | 31.36 | 60.85 |
| 2015CZ | BAd71-2 | 90.05 | 53.23 | 28.72 | 58.54 |
| 2015CZ | BAd128-2 | 81.30 | 58.48 | 37.67 | 55.66 |
| 2015CZ | BAd152-7 | 97.74 | 48.69 | 39.32 | 57.24 |
| 2015CZ | BAd162-5 | 77.06 | 104.05 | 35.82 | 58.89 |
| 2015CZ | BAd182-5 | 66.59 | 83.98 | 34.62 | 54.79 |
| 2015CZ | BAd122-3 | 92.16 | 70.08 | 32.32 | 53.35 |
| 2015CZ | BAd125-2 | 77.49 | 48.87 | 27.18 | 51.53 |
| 2015CZ | BAd169-4 | 56.61 | 65.00 | 33.39 | 56.64 |
| 2015CZ | BAd104-2 | 100.87 | 44.62 | 34.21 | 55.33 |
| 2015CZ | BAd142-5 | 53.26 | 74.70 | 34.52 | 51.67 |
| 2015CZ | BAd164-5 | 86.69 | 46.68 | 30.38 | 52.26 |
| 2015CZ | BAd175-3 | 74.90 | 46.76 | 36.72 | 54.83 |
| 2015CZ | BAd170-4 | 91.28 | 56.00 | 44.21 | 54.65 |
| 2015CZ | BAd182-3 | 112.02 | 59.44 | 41.06 | 51.08 |
| 2015CZ | BAd76-5 | 98.29 | 61.51 | 33.49 | 49.94 |
| 2015CZ | BAd106-4 | 118.52 | 61.33 | 37.82 | 56.91 |
| 2015CZ | BAd124-4 | 84.69 | 67.38 | 35.17 | 52.87 |
| 2015CZ | BAd122-5 | 112.23 | 41.87 | 38.47 | 53.55 |
| 2015CZ | BAd142-4 | 101.08 | 47.86 | 46.21 | 54.90 |
| 2015CZ | BAd137-5 | 73.70 | 46.13 | 40.85 | 57.14 |
| 2015CZ | BAd180-3 | 113.99 | 48.27 | 48.34 | 59.16 |
| 2015CZ | BAd89-1 | 100.65 | 65.67 | 32.47 | 54.76 |
| 2015CZ | BAd179-4 | 75.26 | 56.43 | 30.26 | 54.75 |
| 2015CZ | BAd129-2 | 74.08 | 53.69 | 33.63 | 56.97 |
| 2015CZ | BZn128-5 | 73.48 | 80.52 | 48.59 | 57.26 |
| 2015CZ | BAd112-6 | 95.90 | 52.25 | 30.25 | 50.14 |
| 2015CZ | BAd183-3 | 92.91 | 55.73 | 43.21 | 54.70 |
| 2015CZ | BFe164-6 | 102.41 | 72.14 | 38.57 | 55.22 |
| 2015CZ | AdA79-4 | 60.17 | 72.26 | 34.87 | 50.43 |
| 2015CZ | AdA79-2 | 119.26 | 64.06 | 32.48 | 51.01 |
| 2015CZ | AdA95-1 | 110.32 | 61.69 | 38.53 | 51.21 |
| 2015CZ | AdA168-(1) | 112.87 | 63.86 | 36.15 | 53.85 |
| 2015CZ | AdA168-(16) | 96.14 | 55.00 | 27.35 | 52.57 |
| 2015CZ | AdA168-(20) | 62.10 | 64.73 | 30.94 | 49.39 |
| 2015CZ | AdA168-1 | 94.06 | 61.10 | 33.74 | 64.63 |
| 2015CZ | AdA168-4 | 71.41 | 52.77 | 35.62 | 53.18 |
| 2015CZ | AdA73-8 | 55.08 | 53.59 | 29.27 | 53.59 |
| 2015CZ | AdA73-3 | 73.39 | 58.12 | 29.64 | 53.96 |
| 2015CZ | AdA73-6 | 75.39 | 43.63 | 30.53 | 51.90 |
| 2015CZ | AdA73-11 | 59.14 | 58.52 | 32.38 | 51.92 |
| 2015CZ | AdA73-10 | 78.54 | 60.52 | 31.31 | 49.70 |
| 2015CZ | AdA168-9 | 81.13 | 51.14 | 33.47 | 53.47 |
| 2015CZ | AdA168-5 | 54.74 | 58.46 | 30.84 | 50.57 |
| 2015CZ | AdA168-6 | 86.86 | 65.51 | 30.39 | 53.58 |
| 2015CZ | AdA73-9 | 69.93 | 64.79 | 40.74 | 52.62 |
| 2015CZ | BAd63-4 | 91.04 | 70.21 | 34.98 | 55.90 |
| 2015CZ | AdA73-1 | 84.64 | 51.11 | 27.37 | 52.29 |
| 2015CZ | AdA168-2 | 63.97 | 54.79 | 27.36 | 52.77 |
| 2015CZ | AdA168-(17) | 69.82 | 55.15 | 26.05 | 51.42 |
| 2015CZ | AdA168-(10) | 55.80 | 56.51 | 27.58 | 50.87 |
| 2015CZ | AdA168-(3) | 105.36 | 55.42 | 32.84 | 50.11 |
| 2015CZ | AdA168-(4) | 78.64 | 52.62 | 30.37 | 50.05 |
| 2015CZ | AdA168-(5) | 73.18 | 57.57 | 29.93 | 53.41 |
| 2015CZ | AdA168-(2) | 75.20 | 58.63 | 28.63 | 49.95 |
| 2015CZ | AdA168-(21) | 93.12 | 58.28 | 28.35 | 52.67 |
| 2015CZ | AdA168-(11) | 77.38 | 55.20 | 35.16 | 52.00 |
| 2015CZ | AdA168-(18) | 95.27 | 62.15 | 35.75 | 53.89 |
| 2015CZ | AdA168-(8) | 78.68 | 44.99 | 24.17 | 51.32 |
| 2015CZ | AdA168-(12) | 74.76 | 42.75 | 27.57 | 52.51 |
| 2015CZ | AdA168-(19) | 74.90 | 53.32 | 33.14 | 50.73 |
| 2015CZ | AdA73-(3) | 65.09 | 45.40 | 30.46 | 54.03 |
| 2015CZ | AdA73-(4) | 113.74 | 60.10 | 28.61 | 50.36 |
| 2015CZ | AdA73-(2) | 83.52 | 47.48 | 28.93 | 54.87 |
| 2015CZ | AdA116-(5) | 51.80 | 47.70 | 24.85 | 55.43 |
| 2015CZ | AdA116-(3) | 72.10 | 43.25 | 29.27 | 53.93 |
| 2015CZ | AdA116-⑩ | 69.95 | 53.43 | 30.32 | 55.68 |
| 2015CZ | AdA116-(6) | 107.84 | 48.87 | 33.21 | 52.78 |
| 2015CZ | AdA116-(4) | 50.15 | 43.15 | 28.64 | 51.18 |
| 2015CZ | AdA180-3 | 71.47 | 49.47 | 30.17 | 49.34 |
| 2015CZ | BAd106-1 | 65.13 | 53.02 | 32.12 | 56.97 |
| 2015CZ | BAd142-6 | 65.90 | 86.58 | 30.75 | 50.10 |
| 2015CZ | BAd159-3 | 68.90 | 58.59 | 27.92 | 59.66 |
| 2015CZ | BAd183-4 | 73.21 | 52.16 | 35.07 | 52.54 |
| 2016WJ | CN16 | 47.51 | 37.07 | 27.03 | 49.22 |
| 2016WJ | BAd160-6 | 70.35 | 56.98 | 31.20 | 53.33 |
| 2016WJ | BAd70-2 | 142.71 | 49.61 | 30.30 | 53.44 |
| 2016WJ | BAd71-2 | 139.48 | 90.74 | 30.10 | 52.00 |
| 2016WJ | BAd128-2 | 137.92 | 64.63 | 37.14 | 55.56 |
| 2016WJ | BAd152-7 | 152.30 | 82.47 | 40.24 | 55.11 |
| 2016WJ | BAd162-5 | 145.77 | 66.66 | 34.26 | 57.56 |
| 2016WJ | BAd182-5 | 145.31 | 66.58 | 35.09 | 50.10 |
| 2016WJ | BAd122-3 | 138.09 | 66.00 | 34.24 | 51.00 |
| 2016WJ | BAd169-4 | 138.59 | 78.83 | 35.17 | 51.56 |
| 2016WJ | BAd164-5 | 132.84 | 54.31 | 29.60 | 56.44 |
| 2016WJ | BAd175-3 | 150.61 | 73.96 | 37.48 | 50.89 |
| 2016WJ | BAd170-4 | 139.57 | 75.80 | 42.69 | 56.44 |
| 2016WJ | BAd182-3 | 143.34 | 79.80 | 41.23 | 53.22 |
| 2016WJ | BAd76-5 | 133.05 | 60.55 | 32.81 | 50.67 |
| 2016WJ | BAd129-4 | 61.65 | 65.57 | 32.81 | 50.89 |
| 2016WJ | BAd144-6 | 148.26 | 71.48 | 42.82 | 50.22 |
| 2016WJ | BAd122-5 | 146.39 | 67.57 | 39.90 | 52.78 |
| 2016WJ | BAd137-5 | 134.87 | 92.61 | 39.46 | 51.56 |
| 2016WJ | BAd180-3 | 139.26 | 73.09 | 51.35 | 57.33 |
| 2016WJ | BAd107-1 | 78.33 | 70.90 | 36.44 | 52.33 |
| 2016WJ | BAd89-1 | 136.25 | 72.00 | 34.43 | 53.22 |
| 2016WJ | BAd179-4 | 127.09 | 57.74 | 30.22 | 54.67 |
| 2016WJ | BFe164-6 | 136.38 | 81.13 | 37.65 | 51.89 |
| 2016WJ | AdA79-2 | 108.44 | 59.04 | 31.89 | 49.33 |
| 2016WJ | AdA95-2 | 86.73 | 60.83 | 34.16 | 50.00 |
| 2016WJ | AdA95-1 | 93.62 | 84.84 | 37.80 | 51.22 |
| 2016WJ | BAd149-3 | 59.49 | 55.43 | 32.12 | 52.33 |
| 2016WJ | BAd143-3 | 73.68 | 58.02 | 27.60 | 50.26 |
| 2016WJ | AdA168-1 | 81.23 | 86.76 | 30.92 | 51.00 |
| 2016WJ | AdA168-4 | 66.60 | 71.55 | 32.58 | 53.44 |
| 2016WJ | AdA73-8 | 61.45 | 77.90 | 27.34 | 49.78 |
| 2016WJ | AdA168-9 | 66.72 | 57.85 | 32.35 | 53.89 |
| 2016WJ | AdA168-5 | 59.76 | 68.64 | 28.71 | 50.67 |
| 2016WJ | AdA168-6 | 67.11 | 72.75 | 29.32 | 50.67 |
| 2016WJ | AdA73-9 | 63.12 | 82.26 | 39.84 | 49.56 |
| 2016WJ | BAd63-4 | 84.42 | 75.86 | 32.59 | 51.22 |
| 2016WJ | AdA168-2 | 70.11 | 66.43 | 28.48 | 52.67 |
| 2016WJ | BAd178-6 | 70.13 | 65.89 | 37.25 | 54.11 |
| 2016WJ | AdA168-(6) | 86.50 | 85.03 | 33.93 | 50.11 |
| 2016WJ | AdA168-(14) | 64.74 | 69.40 | 30.26 | 49.67 |
| 2016WJ | BAd168-3 | 95.04 | 92.83 | 35.47 | 51.24 |
| 2016WJ | AdA168-(5) | 77.46 | 74.40 | 28.59 | 52.11 |
| 2016WJ | AdA168-(21) | 84.25 | 70.33 | 29.32 | 50.67 |
| 2016WJ | AdA168-(11) | 64.69 | 63.03 | 34.82 | 49.67 |
| 2016WJ | AdA168-(18) | 89.94 | 73.11 | 34.33 | 51.78 |
| 2016WJ | AdA73-(3) | 61.25 | 52.86 | 31.16 | 54.00 |
| 2016WJ | AdA73-(4) | 78.01 | 49.20 | 28.81 | 50.00 |
| 2016WJ | AdA116-(3) | 66.93 | 48.81 | 28.86 | 49.44 |
| 2016WJ | BAd167-6 | 82.72 | 72.82 | 37.27 | 51.78 |
| 2016WJ | BAd127-1 | 100.74 | 77.58 | 33.32 | 53.56 |
| 2016WJ | BAd177-2 | 90.00 | 67.20 | 35.15 | 53.44 |
| 2016WJ | BAd141-6 | 88.77 | 77.83 | 32.66 | 50.22 |
| 2016WJ | BAd123-2 | 96.11 | 71.14 | 30.85 | 52.11 |
| 2016WJ | BAd70-4 | 87.48 | 59.75 | 32.39 | 50.56 |
| 2016WJ | BAd161-5 | 96.93 | 66.89 | 34.37 | 53.78 |
| 2016WJ | BAd177-6 | 117.50 | 65.12 | 37.17 | 50.56 |
| 2016WJ | BAd117-3 | 96.66 | 76.47 | 36.23 | 49.44 |
| 2016WJ | BAd133-5 | 106.07 | 73.42 | 30.16 | 49.67 |
| 2016WJ | BAd181-1 | 108.91 | 83.65 | 38.29 | 53.56 |
| 2016WJ | BAd156-2 | 113.62 | 66.31 | 31.17 | 51.44 |
| 2016WJ | BAd180-5 | 102.92 | 62.19 | 33.96 | 51.67 |
| 2016WJ | BAd176-4 | 117.87 | 83.96 | 42.19 | 53.78 |
| 2016WJ | BAd139-6 | 102.16 | 78.12 | 39.95 | 50.22 |
| 2016WJ | BAd175-6 | 109.55 | 67.40 | 34.09 | 49.44 |
| 2016WJ | BAd146-4 | 119.88 | 104.29 | 39.47 | 56.44 |
| 2016WJ | BAd95-4 | 113.97 | 68.79 | 35.72 | 50.33 |
| 2016WJ | BAd170-1 | 116.71 | 85.40 | 35.72 | 51.67 |
| 2016WJ | BAd137-4 | 116.13 | 69.51 | 33.47 | 56.78 |
| 2016WJ | BAd170-6 | 76.32 | 61.73 | 33.03 | 52.11 |
| 2016WJ | BAd134-3 | 132.16 | 64.94 | 29.85 | 54.00 |
| 2016WJ | BAd144-1 | 121.67 | 65.28 | 32.39 | 57.11 |
| 2016WJ | BAd145-3 | 105.20 | 64.49 | 37.45 | 52.37 |
| 2016WJ | BAd74-2 | 63.93 | 57.41 | 30.31 | 53.11 |
| 2016WJ | BAd100-3 | 136.78 | 47.28 | 28.38 | 56.56 |
| 2016WJ | BAd163-2 | 79.25 | 74.57 | 45.99 | 53.33 |
| 2016WJ | BAd124-6 | 129.49 | 71.57 | 35.89 | 51.67 |
| 2016WJ | BAd106-1 | 75.20 | 66.36 | 32.80 | 56.33 |
| 2016WJ | BZn130-3 | 75.66 | 66.48 | 34.46 | 50.22 |
| 2016WJ | BAd159-3 | 72.80 | 78.25 | 27.31 | 60.11 |
| 2016WJ | BAd183-4 | 86.18 | 66.77 | 37.46 | 56.78 |
| 2016WJ | BAd173-5 | 124.65 | 68.83 | 38.06 | 50.67 |
| 2016WJ | BAd175-7 | 160.51 | 77.88 | 39.55 | 51.00 |
| 2016WJ | BAd111-1 | 79.41 | 111.28 | 42.43 | 55.56 |
| 2016WJ | BAd178-2 | 133.98 | 71.86 | 38.45 | 50.67 |
| 2016WJ | BAd170-2 | 138.51 | 81.75 | 44.20 | 53.89 |
| 2016WJ | BAd109-2 | 115.80 | 70.79 | 42.07 | 55.78 |
| 2016WJ | BAd157-4 | 137.24 | 73.04 | 34.59 | 54.33 |
| 2016CZ | CN16 | 44.19 | 36.67 | 25.57 | 42.70 |
| 2016CZ | BAd95-2 | 114.04 | 55.23 | 32.96 | 44.83 |
| 2016CZ | BAd70-2 | 124.03 | 54.77 | 28.31 | 53.83 |
| 2016CZ | BAd68-2 | 124.75 | 57.28 | 29.73 | 47.74 |
| 2016CZ | BAd71-2 | 105.13 | 75.91 | 28.08 | 54.10 |
| 2016CZ | BAd128-2 | 127.01 | 58.43 | 34.85 | 57.27 |
| 2016CZ | BAd152-7 | 118.44 | 46.72 | 36.00 | 60.88 |
| 2016CZ | BAd162-5 | 101.66 | 82.04 | 30.40 | 53.87 |
| 2016CZ | BAd182-5 | 134.17 | 71.24 | 32.33 | 53.07 |
| 2016CZ | BAd122-3 | 126.90 | 55.74 | 31.84 | 49.69 |
| 2016CZ | BAd108-2 | 122.55 | 66.81 | 33.32 | 47.70 |
| 2016CZ | BAd169-4 | 93.82 | 45.25 | 32.77 | 60.50 |
| 2016CZ | BAd104-2 | 102.90 | 84.34 | 32.58 | 51.53 |
| 2016CZ | BAd142-5 | 141.18 | 65.87 | 32.44 | 43.40 |
| 2016CZ | BAd175-3 | 143.01 | 66.92 | 32.92 | 51.62 |
| 2016CZ | BAd170-4 | 140.59 | 62.63 | 39.64 | 51.38 |
| 2016CZ | BAd182-3 | 122.39 | 57.13 | 37.20 | 51.17 |
| 2016CZ | BAd93-5 | 153.29 | 71.31 | 29.33 | 50.72 |
| 2016CZ | BAd76-5 | 157.01 | 67.90 | 29.42 | 49.83 |
| 2016CZ | BAd129-4 | 77.31 | 54.72 | 30.67 | 52.52 |
| 2016CZ | BAd106-4 | 111.90 | 78.50 | 35.47 | 49.38 |
| 2016CZ | BAd122-5 | 140.87 | 66.00 | 35.76 | 44.78 |
| 2016CZ | BAd142-4 | 105.47 | 64.51 | 44.24 | 45.39 |
| 2016CZ | BAd137-5 | 142.93 | 47.67 | 35.36 | 51.63 |
| 2016CZ | BAd180-3 | 113.35 | 57.91 | 45.34 | 52.39 |
| 2016CZ | BAd119-3 | 105.17 | 59.22 | 30.94 | 52.05 |
| 2016CZ | BAd107-1 | 88.95 | 66.67 | 34.37 | 52.03 |
| 2016CZ | BAd89-1 | 94.88 | 62.79 | 32.25 | 47.88 |
| 2016CZ | BAd73-2 | 108.31 | 61.26 | 28.64 | 45.25 |
| 2016CZ | BAd179-4 | 101.22 | 64.36 | 27.76 | 50.20 |
| 2016CZ | BAd129-2 | 135.53 | 63.18 | 33.65 | 57.15 |
| 2016CZ | BZn128-5 | 84.39 | 90.63 | 45.34 | 47.40 |
| 2016CZ | BAd112-6 | 101.43 | 80.03 | 30.45 | 47.69 |
| 2016CZ | BAd183-3 | 150.44 | 45.00 | 40.35 | 47.47 |
| 2016CZ | BFe164-6 | 146.49 | 75.12 | 33.56 | 46.84 |
| 2016CZ | AdA95-5 | 104.18 | 82.45 | 30.65 | 46.99 |
| 2016CZ | AdA79-1 | 101.58 | 87.00 | 32.45 | 50.60 |
| 2016CZ | AdA95-3 | 90.95 | 54.94 | 26.67 | 47.13 |
| 2016CZ | AdA79-5 | 107.20 | 63.98 | 26.34 | 46.23 |
| 2016CZ | AdA79-4 | 69.80 | 62.68 | 29.20 | 54.01 |
| 2016CZ | AdA79-2 | 100.88 | 61.40 | 30.81 | 52.55 |
| 2016CZ | AdA95-2 | 80.34 | 53.53 | 34.05 | 49.13 |
| 2016CZ | AdA95-1 | 105.42 | 69.59 | 32.26 | 50.94 |
| 2016CZ | BAd149-3 | 54.54 | 43.96 | 30.32 | 55.55 |
| 2016CZ | BAd143-3 | 90.80 | 52.44 | 26.87 | 49.11 |
| 2016CZ | AdA168-(1) | 114.39 | 56.68 | 31.68 | 50.80 |
| 2016CZ | AdA168-(20) | 103.53 | 97.58 | 28.74 | 47.10 |
| 2016CZ | AdA168-1 | 109.29 | 68.22 | 27.53 | 54.40 |
| 2016CZ | AdA168-4 | 116.26 | 59.52 | 30.53 | 49.73 |
| 2016CZ | BAd71-3 | 90.76 | 54.05 | 32.85 | 45.83 |
| 2016CZ | AdA73-10 | 66.06 | 57.95 | 28.27 | 51.21 |
| 2016CZ | AdA168-9 | 58.50 | 57.09 | 31.10 | 53.85 |
| 2016CZ | AdA168-6 | 61.43 | 66.85 | 27.32 | 46.74 |
| 2016CZ | AdA73-9 | 66.36 | 60.01 | 36.44 | 45.37 |
| 2016CZ | BAd64-5 | 84.78 | 49.85 | 31.71 | 45.17 |
| 2016CZ | BAd63-4 | 68.42 | 64.96 | 29.56 | 47.23 |
| 2016CZ | BAd178-6 | 101.04 | 72.13 | 34.79 | 52.48 |
| 2016CZ | AdA168-(6) | 83.55 | 66.78 | 30.54 | 45.70 |
| 2016CZ | AdA168-(3) | 95.91 | 54.62 | 28.72 | 46.97 |
| 2016CZ | BAd168-3 | 91.90 | 53.07 | 33.78 | 50.83 |
| 2016CZ | AdA168-(5) | 71.97 | 66.34 | 26.85 | 44.09 |
| 2016CZ | AdA168-(21) | 67.96 | 66.00 | 28.05 | 49.05 |
| 2016CZ | AdA168-(11) | 99.25 | 58.65 | 30.38 | 50.06 |
| 2016CZ | AdA168-(12) | 90.77 | 48.38 | 27.31 | 49.99 |
| 2016CZ | AdA168-(19) | 100.29 | 67.73 | 29.40 | 43.70 |
| 2016CZ | AdA73-(3) | 66.26 | 50.46 | 30.67 | 43.31 |
| 2016CZ | AdA116-⑩ | 64.60 | 67.08 | 30.75 | 44.15 |
| 2016CZ | AdA116-(6) | 80.82 | 44.60 | 26.87 | 45.68 |
| 2016CZ | BAd167-6 | 93.92 | 63.88 | 34.11 | 50.00 |
| 2016CZ | BAd127-1 | 96.66 | 53.08 | 30.74 | 46.78 |
| 2016CZ | BAd177-2 | 93.72 | 99.91 | 32.17 | 53.78 |
| 2016CZ | BAd141-6 | 80.34 | 49.14 | 29.88 | 47.67 |
| 2016CZ | BAd123-2 | 95.13 | 54.03 | 29.92 | 51.87 |
| 2016CZ | BAd70-4 | 86.90 | 49.27 | 30.73 | 50.11 |
| 2016CZ | BAd147-2 | 97.22 | 64.16 | 33.14 | 48.10 |
| 2016CZ | BAd161-5 | 114.08 | 63.08 | 30.14 | 50.09 |
| 2016CZ | BAd73-7 | 95.45 | 49.71 | 27.97 | 46.20 |
| 2016CZ | BAd177-6 | 92.61 | 61.18 | 31.58 | 49.33 |
| 2016CZ | BAd133-5 | 103.78 | 65.65 | 29.70 | 50.56 |
| 2016CZ | BAd181-1 | 97.14 | 59.45 | 35.39 | 48.00 |
| 2016CZ | BAd67-2 | 101.19 | 53.46 | 26.03 | 47.56 |
| 2016CZ | BAd156-2 | 125.69 | 62.54 | 30.06 | 51.00 |
| 2016CZ | BAd185-1 | 103.59 | 52.93 | 37.80 | 45.67 |
| 2016CZ | BAd153-3 | 93.50 | 60.76 | 32.12 | 50.89 |
| 2016CZ | BAd180-5 | 81.93 | 53.43 | 30.34 | 53.89 |
| 2016CZ | BAd176-4 | 93.89 | 58.23 | 37.66 | 53.58 |
| 2016CZ | BAd139-6 | 109.11 | 69.89 | 36.43 | 50.89 |
| 2016CZ | BAd175-6 | 119.31 | 68.18 | 30.98 | 54.06 |
| 2016CZ | BAd146-4 | 99.95 | 63.81 | 35.25 | 55.51 |
| 2016CZ | BAd95-4 | 104.49 | 58.58 | 31.63 | 49.40 |
| 2016CZ | BAd170-1 | 94.20 | 61.56 | 33.21 | 55.56 |
| 2016CZ | BAd79-5 | 83.19 | 55.84 | 32.54 | 44.78 |
| 2016CZ | BAd137-4 | 108.67 | 56.55 | 30.86 | 49.67 |
| 2016CZ | BAd170-6 | 95.15 | 51.46 | 31.90 | 51.75 |
| 2016CZ | BAd97-2 | 81.70 | 39.61 | 34.14 | 51.70 |
| 2016CZ | BAd134-3 | 100.49 | 52.41 | 27.69 | 51.31 |
| 2016CZ | BAd144-1 | 104.57 | 53.59 | 30.62 | 55.02 |
| 2016CZ | BAd84-5 | 90.75 | 57.12 | 33.21 | 52.36 |
| 2016CZ | BAd107-4 | 76.25 | 66.32 | 30.43 | 48.44 |
| 2016CZ | BAd128-6 | 92.10 | 62.27 | 26.02 | 51.62 |
| 2016CZ | BAd145-3 | 116.09 | 51.93 | 33.18 | 49.47 |
| 2016CZ | BAd74-2 | 85.23 | 52.58 | 27.66 | 49.90 |
| 2016CZ | BAd100-3 | 126.28 | 67.49 | 28.06 | 42.93 |
| 2016CZ | BAd163-2 | 104.88 | 58.01 | 41.77 | 52.72 |
| 2016CZ | BAd124-6 | 148.01 | 100.71 | 32.99 | 44.10 |
| 2016CZ | BAd106-1 | 82.16 | 56.55 | 30.67 | 53.00 |
| 2016CZ | BZn130-3 | 91.56 | 51.80 | 31.05 | 51.40 |
| 2016CZ | BAd142-6 | 69.36 | 59.62 | 28.38 | 50.64 |
| 2016CZ | BAd159-3 | 72.02 | 63.02 | 26.68 | 60.75 |
| 2016CZ | BAd183-4 | 93.83 | 48.14 | 32.32 | 50.91 |
| 2016CZ | BAd173-5 | 97.75 | 65.21 | 35.39 | 51.07 |
| 2016CZ | BAd175-7 | 115.68 | 60.87 | 34.34 | 49.39 |
| 2016CZ | BAd111-1 | 103.16 | 69.10 | 37.83 | 53.87 |
| 2016CZ | BAd178-2 | 133.30 | 58.05 | 35.00 | 49.00 |
| 2016CZ | BAd69-2 | 132.22 | 58.45 | 30.82 | 43.21 |
| 2016CZ | BAd170-2 | 153.37 | 91.41 | 41.60 | 49.29 |
| 2016CZ | BAd109-2 | 126.06 | 62.51 | 38.18 | 47.03 |
| 2016CZ | BAd157-4 | 104.85 | 55.06 | 30.41 | 56.31 |

**Supplementary Table 2.** The Phenotypic values of GFeC, GZnC, GMnC and TKW in the top ranking 61 wheat lines derived from wild emmer and their parents based on BLUP data.

| Traits | Parents | | Advanced lines | |
| --- | --- | --- | --- | --- |
|  | CN16 | D1 | Average | Range |
| GFeC mg/kg | 58.34 | 101.77 | 100.62*** | 62.73-125.58 |
| GZnC mg/kg | 44.60 | 79.27 | 60.74*** | 47.00-75.09 |
| GMnC mg/kg | 25.53 | 33.39 | 34.56*** | 21.71-49.50 |
| TKW g | 46.80 | 30.32 | 49.54*** | 42.64-53.87 |

Note: significantly different from CN16 at P<0.001(***).

**Supplementary Table 3.** Potential candidate genes containing/flanking MTAs for improving grain micronutrients in wheat lines derived from wild emmer wheat.

| Trait | MTA | Chr. | PVE (%) | | IWGSC v1.0 database | | Wild emmer database | |
| --- | --- | --- | --- | --- | --- | --- | --- | --- |
|  |  |  | MLM | GLM | Gene ID | Gene Annotation | Gene ID | Gene Annotation |
| GFeC | 1210301 | 3B | 8.10 | 8.44 | \ | \ | TRIDC3BG000820 | beta glucosidase 43 |
|  |  |  |  |  |  |  | TRIDC3BG000840 | HD domain-containing metal-dependent phosphohydrolase family protein |
|  | 2255722 | 4A | 10.45 | 17.12 | TraesCS4A01G105400.1 | Hydrolase family protein | TRIDC4AG071440.2 | Kelch repeat-containing protein |
|  |  |  |  |  | TraesCS4A01G105500.1 | Thioredoxin-like protein AAED1 | TRIDC4AG071450.1 | unknown function |
|  |  |  |  |  | \ | \ | TRIDC4AG071460 | receptor-like protein kinase 4 |
|  |  |  |  |  |  |  | TRIDC4AG071290 | Leucine-rich repeat-containing protein 57 |
|  | 3024845 | 4B | 8.31 | 17.42 | TraesCS4B01G549700LC.1 | MYB transcription factor | TRIDC4BG057930 | MYB-related transcription factor |
|  |  |  |  |  | TraesCS4B01G344800.1 | iron-dependent dioxygenase | TRIDC4BG058030 | unknown function |
|  |  |  |  |  | TraesCS4B01G550900LC.1 | FAR1-related sequence 5 | \ | \ |
|  | 2258533/3033960 | 5A | 7.85/9.47 | 18.70/14.81 | TraesCS5A01G001100 | Sugar transporter | TRIDC5AG000480 | sugar transporter 1 |
|  |  |  |  |  | TraesCS5A01G001500 | Carotenoid cleavage dioxygenase | TRIDC5AG000420 | carotenoid cleavage dioxygenase 1 |
|  |  |  |  |  | TraesCS5A01G000700 | Metal-dependent hydrolase | TRIDC5AG000430 | FAR1-related sequence 3 |
|  | 1274451 | 7B | 7.18 | 7.49 | TraesCS7B01G448400LC.1 | catalytic LigB subunit of aromatic ring-opening dioxygenase family | TRIDC7BG042040.1 | FAR1-related sequence 5 |
|  |  |  |  |  | TraesCS7B01G449100LC.1 | FAR1-related sequence 3 (zinc ion binding) | \ | \ |
| GZnC | 1136167 | 1A | 7.6 | 8.69 | TraesCS1A01G533200LC.1 | Protein FAR1-related sequence 5 (zinc ion binding) | TRIDC1AG056010 | Zinc finger BED domain-containing protein RICESLEEPER 1 |
|  |  |  |  |  | TraesCS1A01G534300LC.1 | zinc-binding in reverse transcriptase | \ | \ |
|  | 1077698 | 2A | 7.88 | 9.92 | \ | \ | TRIDC2AG051710 | Heavy metal transport |
|  |  |  |  |  |  |  | TRIDC2AG051950 | Zinc finger (C3HC4-type RING finger) family protein |
|  | 1234362 | 2A | 7.86 | 7.7 | TraesCS2A01G259900LC.1 | P-loop containing nucleoside triphosphate hydrolases superfamily protein | \ | \ |
|  |  |  |  |  | TraesCS2A01G260000LC.1 | Mitogen-activated protein kinase (MAPK) | TRIDC2AG030250 | unknown function |
| GMnC | 3023738/2326413 | 1B | 11.04/11.84 | 7.53/7.81 | TRIDC1BG025600 | ABC transporter family protein | TRIDC1BG024520.1 | undescribed protein |
|  | 1114828 | 1B | 8.16 | 6.57 | TraesCS1B01G367200.1 | Transmembrane protein | TRIDC1BG041170.1 | unknown function |
|  |  |  |  |  | \ | \ | TRIDC1BG041190 | S-adenosyl-L-methionine-dependent methyltransferases superfamily protein |
|  | 1215559/1105781 | 1B | 8.04/8.98 | 8.43/6.15 | TraesCS1B01G463700LC.1 | phosphatase family protein | TRIDC1BG041990 | Argininosuccinate synthase |
|  |  |  |  |  | TraesCS1B01G463400LC.1 | protein variation in compound triggered root growth protein | \ | \ |

Note: Chr, Chromosomal location information based on the wheat consensus map version 3.0 (http://www.diversityarrays.com/sequence-maps) and the best hit on IWGSC (the International Wheat Genome Sequencing Consortium), respectively. "\", data unavailable.
